# Kinase inhibition of G2019S-LRRK2 enhances autolysosome formation and function to reduce endogenous alpha-synuclein intracellular inclusions

**DOI:** 10.1038/s41420-020-0279-y

**Published:** 2020-06-08

**Authors:** Julia Obergasteiger, Giulia Frapporti, Giulia Lamonaca, Sara Pizzi, Anne Picard, Alexandros A. Lavdas, Francesca Pischedda, Giovanni Piccoli, Sabine Hilfiker, Evy Lobbestael, Veerle Baekelandt, Andrew A. Hicks, Corrado Corti, Peter P. Pramstaller, Mattia Volta

**Affiliations:** 1 Institute for Biomedicine, Eurac Research, Affiliated Institute of the University of Lübeck - Via Galvani 31, 39100 Bolzano, Italy; 2 Department of Cellular, Computational and Integrative Biology-CIBIO, University of Trento, Via Sommarive 9, 38123 Povo, TN Italy; 3 Department of Anesthesiology, Rutgers University - New Jersey Medical School, Medical Science Building, 185 South Orange Avenue, Newark, NJ 07103 USA; 4 Department of Neurosciences, KU Leuven, Herestraat 49 bus 1023, 3000 Leuven, Belgium; 5 Department of Neurology, General Central Hospital, Via Böhler 5, 39100 Bolzano, Italy; 6 Department of Neurology, University of Lübeck, Ratzeburger Allee 160, 23538 Lübeck, Germany

**Keywords:** Macroautophagy, Cellular neuroscience

## Abstract

The Parkinson’s disease (PD)-associated kinase Leucine-Rich Repeat Kinase 2 (LRRK2) is a crucial modulator of the autophagy-lysosome pathway, but unclarity exists on the precise mechanics of its role and the direction of this modulation. In particular, LRRK2 is involved in the degradation of pathological alpha-synuclein, with pathogenic mutations precipitating neuropathology in cellular and animal models of PD, and a significant proportion of LRRK2 patients presenting Lewy neuropathology. Defects in autophagic processing and lysosomal degradation of alpha-synuclein have been postulated to underlie its accumulation and onset of neuropathology. Thus, it is critical to obtain a comprehensive knowledge on LRRK2-associated pathology. Here, we investigated a G2019S-LRRK2 recombinant cell line exhibiting accumulation of endogenous, phosphorylated alpha-synuclein. We found that G2019S-LRRK2 leads to accumulation of LC3 and abnormalities in lysosome morphology and proteolytic activity in a kinase-dependent fashion, but independent from constitutively active Rab10. Notably, LRRK2 inhibition was ineffective upon upstream blockade of autophagosome-lysosome fusion events, highlighting this step as critical for alpha-synuclein clearance.

## Introduction

Parkinson’s disease (PD) linked to *Lrrk2* gene mutations is clinically indistinguishable from idiopathic PD (iPD) but with pleomorphic pathology^1^. The G2019S mutation is the most common mutation, with an incidence up to 40% in specific populations^2,3^, and often presents with alpha-synuclein (aSyn) Lewy neuropathology^4^, apart from Tau pathology^5^.

Leucine-Rich Repeat Kinase 2 (LRRK2) is a large multidomain protein with GTPase and kinase domains in close vicinity^6^. The PD-linked mutations reside in this enzymatic core, with G2019S located in the kinase domain, and increase kinase activity^7^.

LRRK2 cellular roles are varied, with stronger consensus on synaptic transmission^8^, vesicle trafficking^9^ and autophagy^10^, which converge in neuronal biology and function^11^.

Several independent investigations demonstrated that LRRK2 acts at different stages of the autophagy-lysosome pathway (ALP), with some conflicting results on the net physiological direction^10^. Indications include a kinase-dependent role in basal autophagy, with studies showing either enhancement or repression^12–15^, and modulation of lysosome function^16,17^. In addition, LRRK2 phosphorylates the small GTPases Rab8a and Rab10 to affect intracellular vesicle dynamics^18^ and decrease fusion between late endosomes and lysosomes via Rab7^19^. Understanding the impact of PD-linked mutations has further increased the complexity of this problem. Most studies indicate aberrant autophagic function induced by mutant LRRK2, including impairment of chaperone-mediated autophagy (CMA) and aSyn processing^20,21^. However, macroautophagy is also altered leading to detrimental cellular consequences^22–24^. Moreover, pathogenic LRRK2 directly reduces lysosome function in different cell types^25–28^. Thus, despite an agreement on LRRK2 playing a role in the ALP, and that PD-linked mutations alter this process, no evidence to date indicates the precise mechanisms.

LRRK2 mediates accumulation of pathologic pS129-aSyn^29,30^ with kinase inhibitors being beneficial against neuropathology^29^. At the same time, neuropathology has been hypothesized to be a consequence of ALP dysfunction (reviewed in ref. ^31^).

A missing link exists in the attempts to put these pieces together and, to the best of our knowledge, no evidence has been reported indicating how PD-mutant LRRK2 specifically affects the ALP and the direct consequences on endogenous aSyn handling.

Here, we set out to investigate how G2019S-LRRK2 pathogenic kinase activity affects the ALP. We found that mutant LRRK2 alters the processing of autophagosomes and lysosomal activity in a kinase-dependent manner. These defects are paralleled by the accumulation of endogenous pS129-aSyn in intracellular inclusions. Lastly, we demonstrate that the efficacy of LRRK2 inhibition in reducing pathologic aSyn depends on the functional fusion between autophagosomes and lysosomes, indicating that this precise step is responsible for aSyn accumulation, while activation of Rab10 has no observable consequences.

## Results

### Autophagy alterations in G2019S-LRRK2 cells exhibiting pS129-aSyn inclusions

**Fig. 1:**
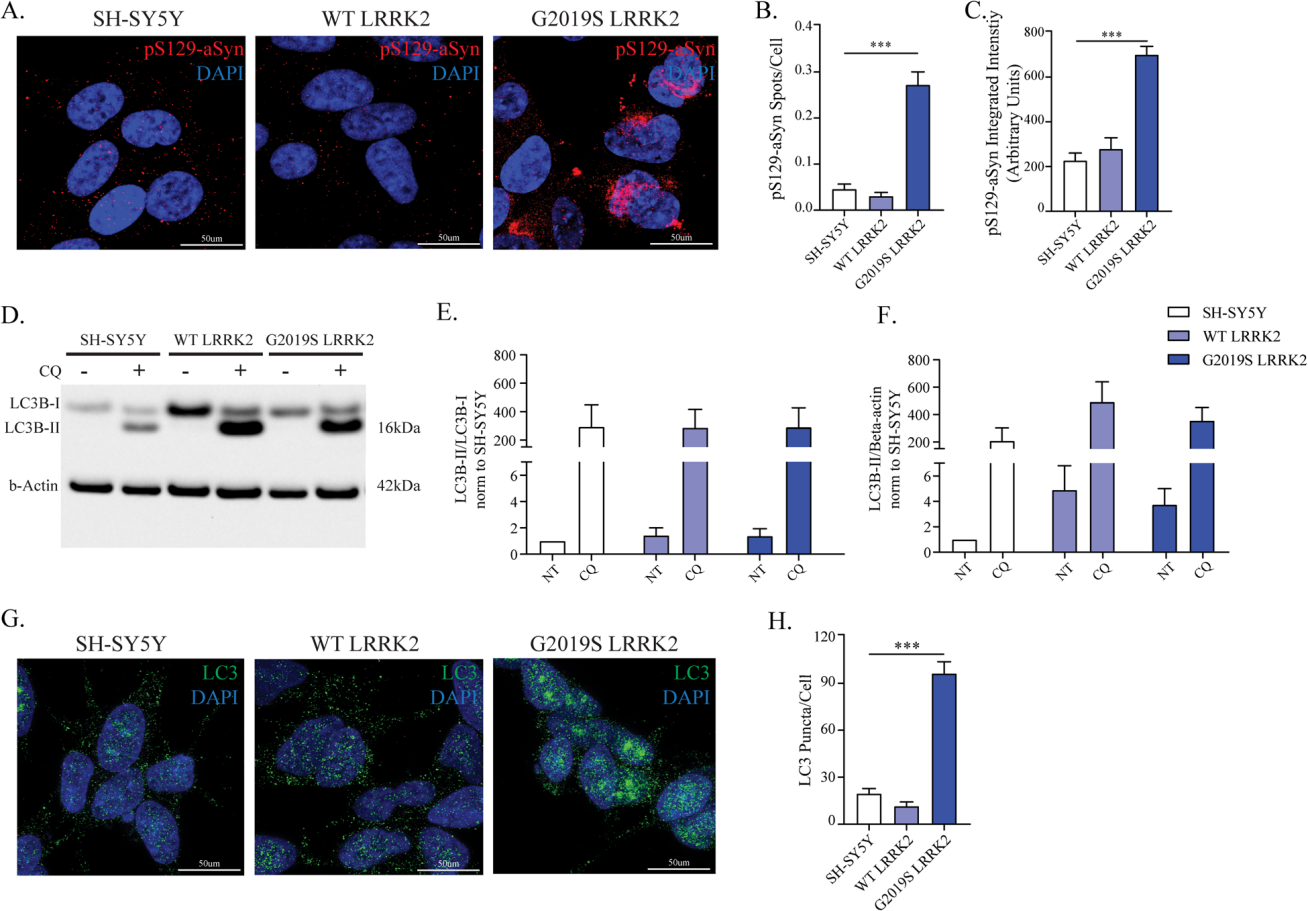
G2019S-LRRK2 cells selectively accumulate intracellular pS129-aSyn inclusions and display LC3 accumulation. **a** Immunocytochemistry for pS129-aSyn was employed to investigate the presence of intracellular inclusions of aSyn. **b** The number of inclusions was quantified and G2019S-LRRK2 cells specifically displayed discrete intracellular objects (*n* = 6). **c** Quantification of integrated intensity of the ICC signal showed a significant increase in G2019S-LRRK2 cells (*n* = 6). **d** The autophagic flux was assessed in WT- and G2019S-LRRK2 cells upon treatment with CQ (100 µM, 3 h) and WB for LC3B. **e** The ratio between LC3B-II and LC3B-I was not different across cell lines or upon CQ treatment, suggesting no differences in autophagic flux (*n* = 3). **f** Quantification of LC3B levels (normalized to β-actin) indicated a strong increase in untreated G2019S-LRRK2 cells, and no differences in CQ-treated cells (*n* = 3). **g** Immunocytochemistry for LC3B was performed to visualize its endogenous distribution and autophagosomes in WT- and G2019S-LRRK2 cells. **h** Quantification of LC3B-positive puncta revealed a significant increase in G2019S-LRRK2 cells (*n* = 4). Data are means ± SEM of 3–7 independent experiments for WB. In imaging experiments, 4–6 independent biological replicates were performed and analysis conducted on 700–1000 cells per group in each experiment. ***p < 0.001, one-way ANOVA followed by the Bonferroni’s post-hoc test.

Confocal imaging of G2019S-LRRK2 cells demonstrates accumulation of endogenous pS129-aSyn resembling cytoplasmic inclusions in cultured cells^32^. In WT-LRRK2, pS129-aSyn signal is weak and diffuse, comparable to control SH-SY5Y cells (Fig. 1a–c). Total aSyn protein levels are not changed between cell lines (Supplementary Fig. S1a, b). Of note, WT-LRRK2 cells display stronger LRRK2 expression than G2019S-LRRK2 ones (Supplementary Fig. S3 and our previous work^33^), suggesting pS129-aSyn accumulation is not solely a direct consequence of enhanced LRRK2 expression.

Since LRRK2 modulates degradation of aSyn by autophagy^10,20^ and its kinase activity is linked to aSyn neuropathology^29^, we reasoned that pS129-aSyn accumulation in our cells could also be related to dysfunctional protein degradation and we interrogated the ALP. A transcriptome analysis screening of 84 ALP-related genes indicated probable ALP alterations in G2019S-LRRK2 cells (Supplementary Table 1). We observed overall slight differences in expression of genes related to lysosome biology (e.g. *CTSD*, *CTSS*), initiation of autophagy (e.g. *MTOR*, *AMBRA1*, *ULK1*) and a strong downregulation of *WIPI1*, previously directly correlated to autophagy dysfunction and reduced autophagosome formation^34^. Then, we assessed autophagic flux by WB to measure the conversion of LC3B-I to LC3B-II upon treatment with CQ (Fig. 1d), which inhibits autolysosome formation^35^. The ratio LC3B-II/LC3B-I was not different in WT- or G2019S-LRRK2 cells, when compared to naïve cells, treated with CQ (Fig. 1e). In vehicle-treated cells, LC3B-II levels (indicative of autophagosome number) strongly trended to an increase in WT- and G2019S-LRRK2, with respect to SH-SY5Y controls (Fig. 1f).

To complement biochemical analyses, we next visualized the pattern distribution of endogenous LC3B and thus turned to immunocytochemistry (Fig. 1g). No difference was detected between naïve and WT-LRRK2 cells, while a strong increase in the number of LC3B puncta per cell was observed in G2019S-LRRK2 (Fig. 1h). Calculation of the Pearson´s coefficient indicated that ~35% of pS129-aSyn colocalized with LC3B (Supplementary Fig. S2a).

In these conditions, cell viability was not affected (Supplementary Fig. S2b), while the growth rate of G2019S-LRRK2 cells is slowed, with respect to WT-LRRK2 (Supplementary Fig. S2c).

These data indicate that LC3B accumulates in G2019S-LRRK2 cells, without an overt increase in autophagosome production, suggesting downstream changes in lysosome biology.

### G2019S-LRRK2 alters lysosome morphology and functionality

**Fig. 2:**
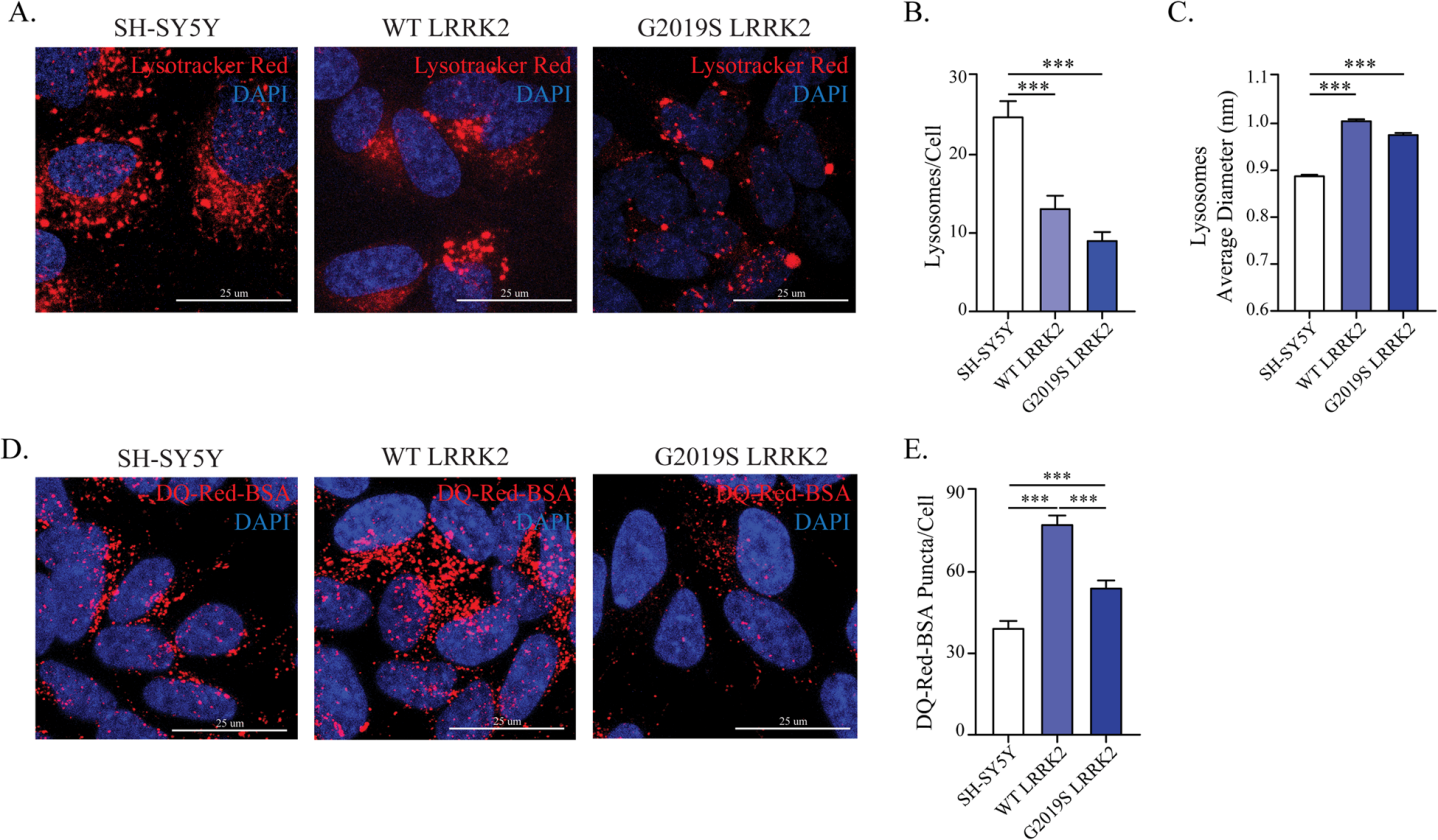
Lysosomal morphology and proteolytic activity are altered in G2019S-LRRK2 cells. **a** Processing of cells with the Lysotracker Red dye was performed to visualize lysosomes in WT- and G2019S-LRRK2 cells. **b** The number of lysosomes per cell was quantified and revealed a decrease in both WT- and G2019S-LRRK2 cell lines (*n* = 4). **c** The average diameter of lysosomes was assessed in parallel. A significant enlargement was observed in both WT- and G2019S-LRRK2 cells (*n* = 4). **d** The DQ-Red-BSA assay was employed to assess the proteolytic activity of lysosomes in WT- and G2019S-LRRK2 cells. **e** Quantification of DQ-Red-BSA fluorescent spots revealed a significant increase in WT-LRRK2 cells, while G2019S-LRRK2 cells displayed significantly fewer spots when compared to WT-LRRK2 cells (*n* = 5). Data are means ± SEM of 4–5 independent experiments and analysis conducted on 700–1000 cells per group in each experiment. ***p < 0.001, one-way ANOVA followed by Bonferroni’s post-hoc test.

The results presented so far indicate that LC3B accumulates in G2019S-LRRK2 cells, but in the absence of an increase in their production. Thus, we used Lysotracker Red to visualize lysosomes as they are the final effector in the ALP (Fig. 2a). In comparison to naïve cells, both WT- and G2019S-LRRK2 cells displayed a significant reduction in the number of lysosomes (Fig. 2b) and a concomitant increase in their size (Fig. 2c). These morphological changes, however, do not inform on the actual degradative capacity of lysosomes. To address this, we next studied the proteolytic activity employing the DQ-Red-BSA assay^36^. The DQ-Red-BSA is endocytosed and trafficked to lysosomes where proteolytic activity triggers fluorescence. Both WT- and G2019S-LRRK2 cells displayed an increase in the number of DQ-Red-BSA puncta, with respect to SH-SY5Y (Fig. 2d, e). However, G2019S-LRRK2 cells had significantly fewer DQ-Red-BSA spots than WT-LRRK2. We conclude that G2019S-LRRK2 produces a defect in lysosomal activity and/or endocytic uptake of DQ-Red-BSA, when compared to WT-LRRK2.

**Fig. 3:**
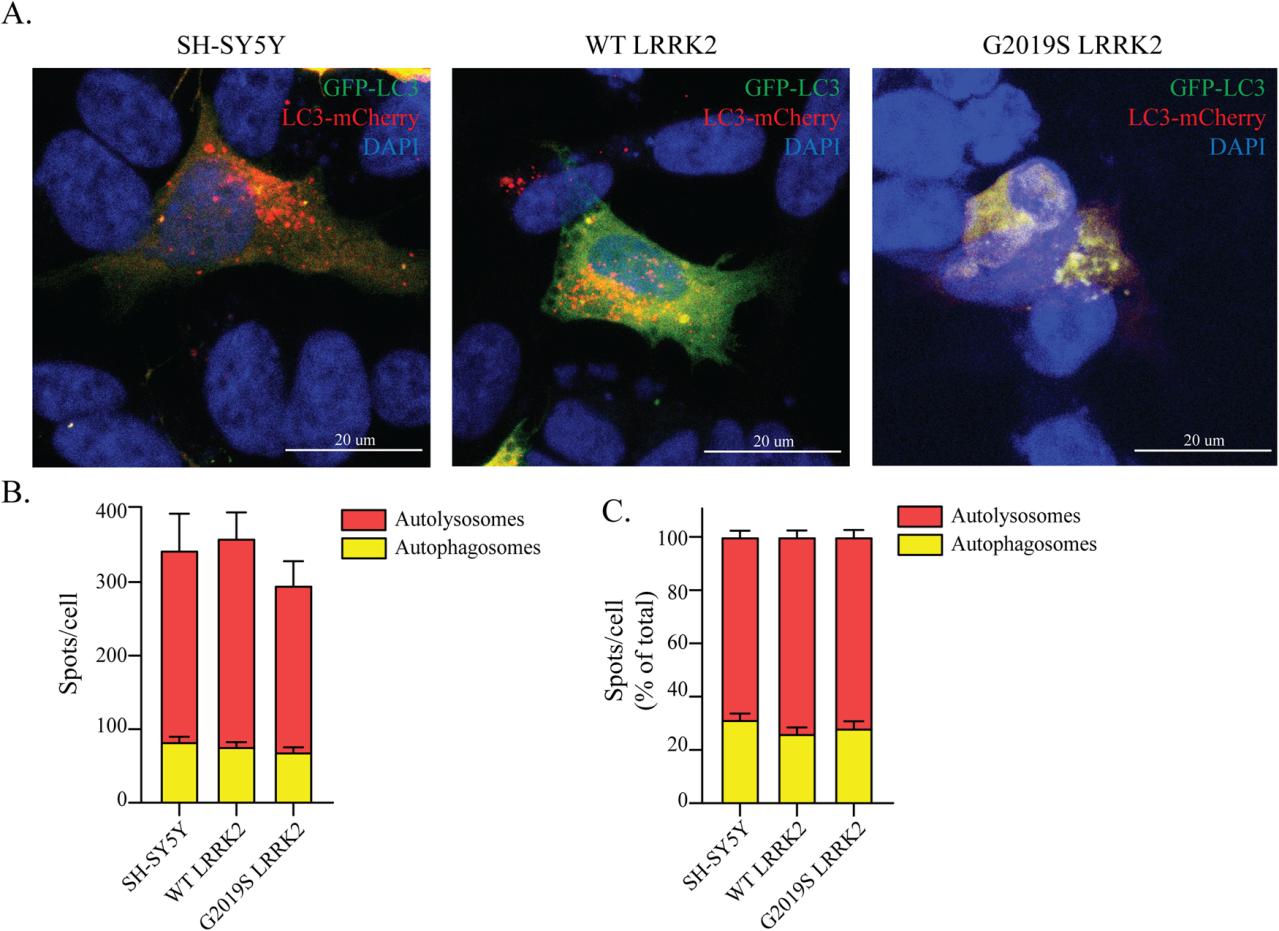
The velocity of the autophagic flux is unchanged in LRRK2 cells. **a** The GFP-LC3-mCherry reporter construct was transfected in WT-, G2019S-LRRK2 and SH-SY5Y control cells and visualized under the confocal microscope to examine the abundance of autophagosomes and autolysosomes. **b** The number of autophagosomes (yellow particles) and autolysosomes (red particles) was determined and revealed no significant differences in our cell lines (*n* = 4). **c** The relative proportion of the autophagic vesicles was also calculated in each cell line (*n* = 4).

Then we sought to more accurately investigate the autophagic flux using the GFP-LC3-mCherry reporter, where the GFP fluorescence is quenched in acidic pH^37^ (see *Supplementary Materials and Methods*). This tool enables a more dynamic approach to ALP study, compared to steady-state protein levels^35^. Using confocal microscopy, we visualized autophagosomes (yellow) and autolysosomes (red) in transfected cells and counted the number of vesicles per cell (Fig. 3a). We detected no differences in the number of vesicles or their percentage representation in all cell lines (Fig. 3b, c).

Altogether, our data indicate that overexpression of G2019S-LRRK2 leads to lysosomal alterations without an induction of autophagy.

### Kinase inhibition of G2019S-LRRK2 reduces aSyn inclusions and LC3B accumulation

The G2019S mutation increases LRRK2 kinase activity^7^ and LRRK2-selective kinase inhibitors have been proposed as therapeutic strategy for PD^38^. We sought to investigate the kinase-dependence of the phenotypes observed in our G2019S-LRRK2 cell line, as these accumulate pS129-aSyn. First, we measured S935 and S1292 phosphorylation^39^ via WB (Supplementary Fig. S3). Expression of endogenous LRRK2 in control SH-SY5Y cells is extremely weak and rendered quantification of optical density highly inaccurate. We observed a strong increase of active LRRK2, measured as pS1292-LRRK2/LRRK2 ratio, in G2019S- when compared to WT-LRRK2 cells, despite a large difference in expression levels (Supplementary Fig. S3a, b). On the other hand, pS935-LRRK2 was not increased (but rather reduced; Supplementary Fig. S3c, d). Then, we employed PF-475 (300 nM–1 µM), which reduced phosphorylation at both residues (Supplementary Fig. S3e)^40,39^ while not affecting cell viability (Supp. Fig. S2d). Given the strong inhibition of pS1292-LRRK2 in G2019S-LRRK2 cells observed at 300 nM, we decided to use 300 nM and 500 nM concentrations in subsequent experiments to avoid protein destabilization^41^. Pharmacological kinase inhibition did not dramatically affect the autophagy-related transcriptome (Supplementary Table 2).

**Fig. 4:**
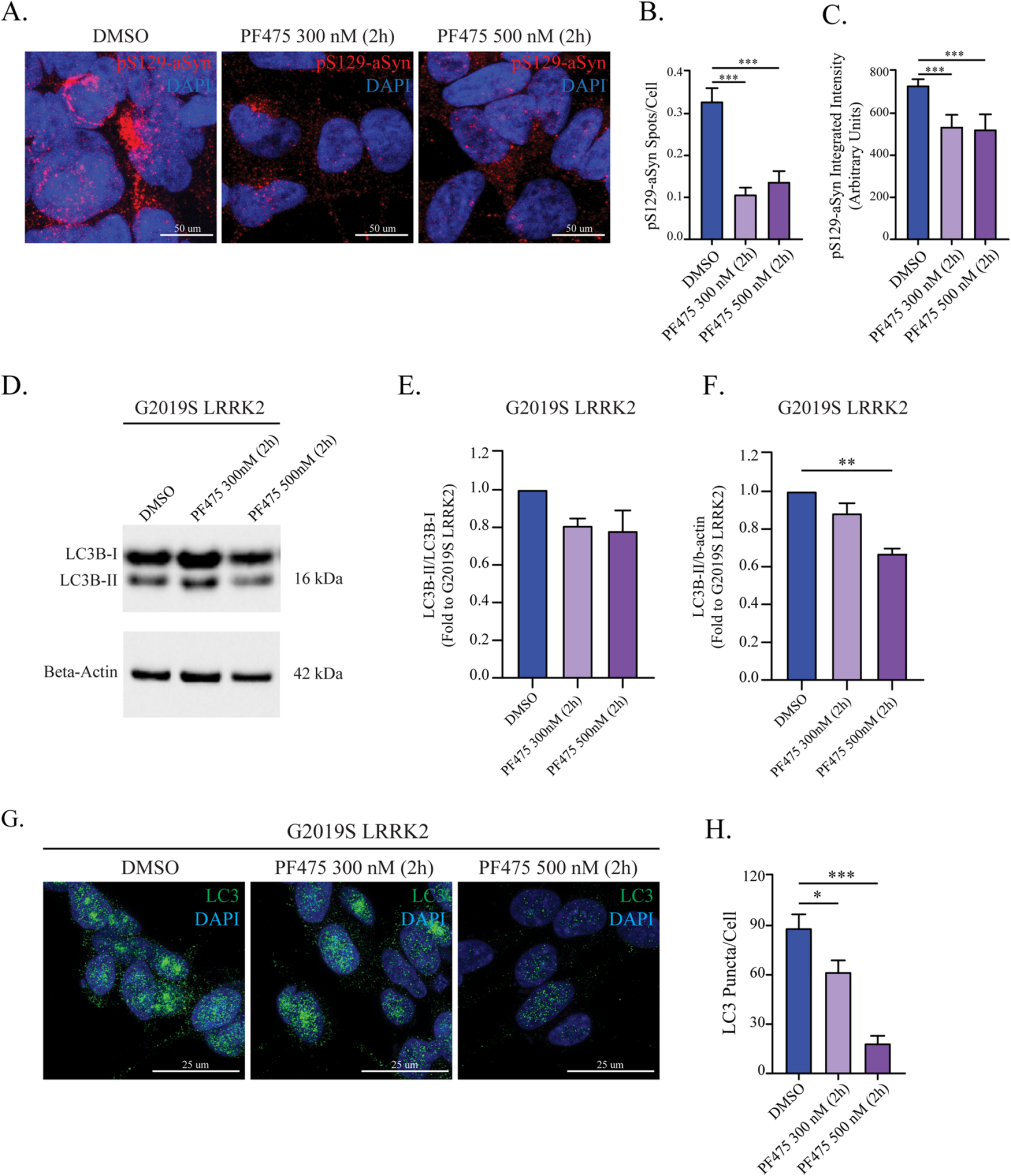
LRRK2 kinase inhibition in G2019S-LRRK2 cells reduces the number of pS129-aSyn inclusions but does not affect autophagic flux. **a** G2019S-LRRK2 cells were treated with PF-475 for 2 h and processed for immunocytochemistry for pS129-aSyn. **b** The number of pS129-aSyn inclusions per cell was quantified and revealed a significant reduction operated by both 300 nM and 500 nM PF-475 (*n* = 4). **c** The integrated intensity of the immunosignal was assessed at the same time and revealed both PF-475 concentrations were effective in reducing pS129-aSyn intensity in G2019S-LRRK2 cells (*n* = 4). **d** G2019S-LRRK2 cells were treated with PF-475 (300 and 500 nM, 2 h) and processed for Western blot for LC3B to evaluate the effect on autophagy initiation. **e** LRRK2 kinase inhibition was not effective on autophagy initiation, as assessed by the conversion of LC3B-I to LC3B-II (*n* = 3). **f** The number of autophagosomes, as assessed by LC3B-II levels, was reduced by PF-475 500 nM in G2019S-LRRK2 cells (*n* = 3). **g** G2019S-LRRK2 cells were treated with PF-475 (2 h) and subjected to immunocytochemistry for LC3B. **h** Quantification of LC3B-positive puncta revealed that PF-475 concentration-dependently reduced the number of puncta in G2019S-LRRK2 cells (*n* = 3). Data are means ± SEM of three independent experiments for WB. Data are means ± SEM of 3–4 independent experiments for immunocytochemistry and analysis conducted on 700–1000 cells per group in each experiment. *p < 0.05, **p < 0.01, ***p < 0.001, one-way ANOVA followed by Bonferroni’s post-hoc test.

Since LRRK2 kinase activity has been shown to mediate aSyn accumulation^29^, we tested whether the abundance of pS129-aSyn inclusions is sensitive to kinase inhibition. We treated G2019S-LRRK2 cells with PF-475 (2 h) and processed for pS129-aSyn immunostaining (Fig. 4a–c). In PF-475-treated cells, pS129-aSyn staining appeared mostly diffuse as opposed to larger structures identified in DMSO control (Fig. 4a). Both 300 nM and 500 nM significantly reduced the number of pS129-aSyn spots (Fig. 4b) and the signal integrated intensity (Fig. 4c). These effects were similar after a 6 h treatment (Supplementary Fig. S4). On the other hand, PF-475 treatment did not affect total aSyn levels (Supplementary Fig S2e). Given that autophagy stimulation has been proposed to combat proteinopathy in neurodegeneration^42^, we assessed the effect of LRRK2 kinase inhibition on LC3B conversion in G2019S-LRRK2 cells as a possible biological substrate underlying the reduction of pS129-aSyn inclusions (Fig. 4d–f). The LC3B-II/LC3B-I ratio was not changed in PF-475-treated cells (Fig. 4d, e), indicating that autophagy initiation is not affected. Conversely, LC3B-II levels (normalized to β-actin) were significantly reduced by 500 nM PF-475 (Fig. 4f), suggesting a decrease in the number of autophagosomes. To substantiate this finding, we applied PF-475 (2 h) before processing the cells for confocal microscopy. Both 300 nM and 500 nM PF-475 significantly reduced the number of LC3B-positive puncta in G2019S-LRRK2 cells (Fig. 4g, h).

Altogether, these data suggest that the accumulation of pS129-aSyn in G2019S-LRRK2 cells depends on its kinase activity.

### LRRK2 kinase inhibition promotes autolysosome formation and lysosomal proteolysis

Our results so far indicate that accumulation of LC3B puncta and pS129-aSyn are mediated by LRRK2 kinase activity. At the same time, autophagic flux and autophagosome production are not affected by the G2019S mutation or the application of PF-475. Thus, both the observed phenotypes and the effect of kinase inhibition are not related to the initial steps of autophagy. Then, to identify the process underlying PF-475-dependent amelioration of aSyn burden, we determined the effects of kinase activity on lysosomal morphology and function.

**Fig. 5:**
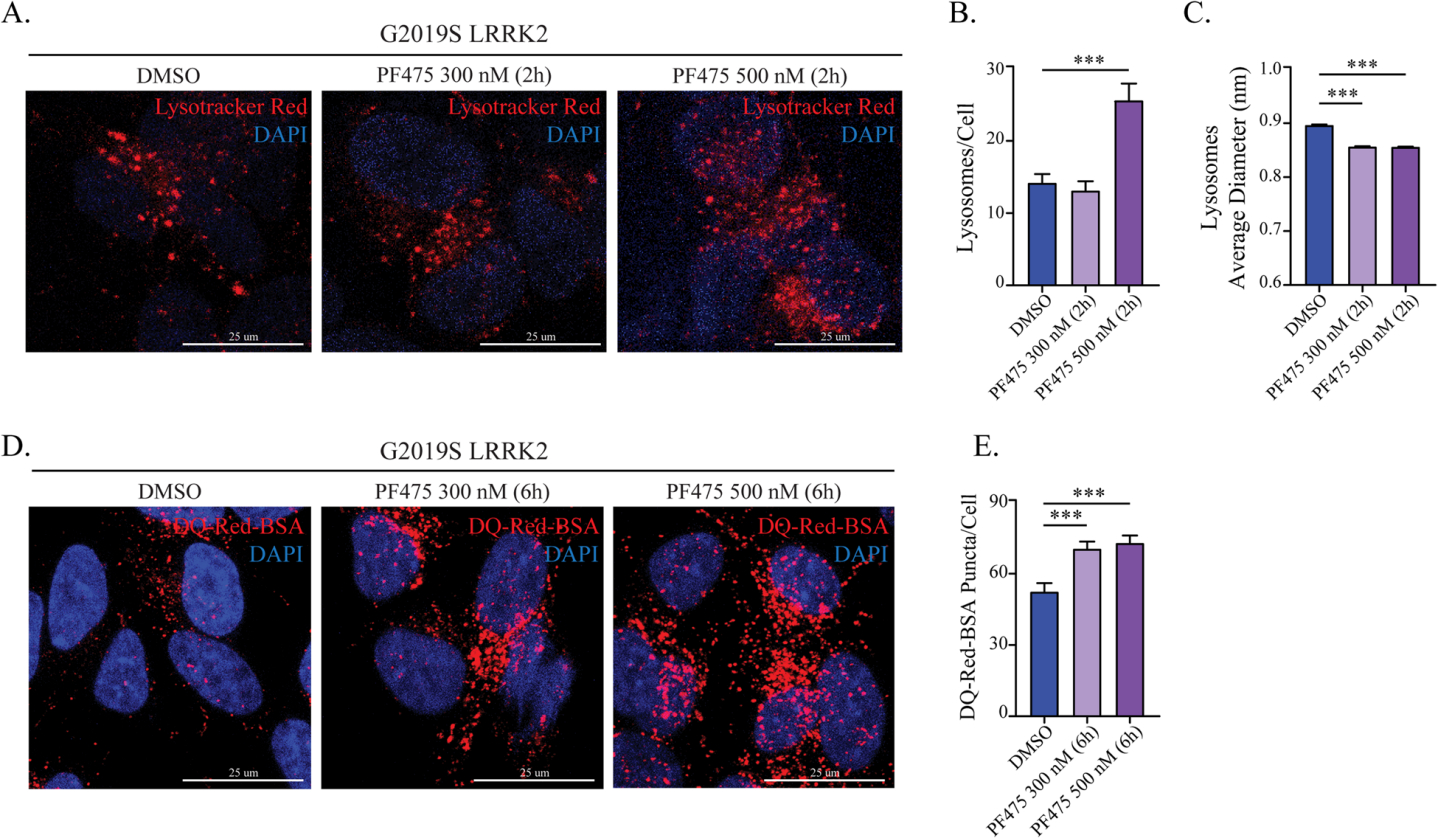
LRRK2 inhibition in G2019S-LRRK2 cells restores lysosome morphology and enhances lysosome activity. **a** G2019S-LRRK2 cells treated with PF-475 were incubated with the Lysotracker Red dye to visualize lysosomes. **b** Quantification of lysosome number per cell revealed that PF-475 500 nM significantly increased their abundance in G2019S-LRRK2 cells (*n* = 3). **c** Determination of lysosome diameter revealed that PF-475, at both concentrations, attenuated the enlargement observed in G2019S-LRRK2 cells (*n* = 3). **d** G2019S-LRRK2 cells were treated with PF-475 for 6 h, then incubated with DQ-Red-BSA and imaged via confocal microscopy to assess proteolytic activity. **e** Quantification of the number of DQ-Red-BSA spots per cell revealed that both concentrations were effective in enhancing lysosome degradative capacity in G2019S-LRRK2 cells (*n* = 4). Data are means ± SEM of 3–4 independent experiments and analysis conducted on 700–1000 cells per group in each experiment. ***p < 0.001, one-way ANOVA followed by Bonferroni’s post-hoc test.

The number of lysosomes per cell was significantly increased by PF-475 (2 h, 500 nM; Fig. 5a, b), while both 300 nM and 500 nM decreased lysosomal size (Fig. 5c).

Using DQ-Red-BSA, we observed that LRRK2 kinase inhibition also affected lysosomal activity (Fig. 5d). Here, we applied PF-475 for 6 h to compensate for the necessary 2 h incubation with DQ-Red-BSA. Both concentrations significantly enhanced the number of fluorescent spots per cell (Fig. 5e), indicating that kinase inhibition enhanced the proteolytic activity in G2019S-LRRK2 cells.

**Fig. 6:**
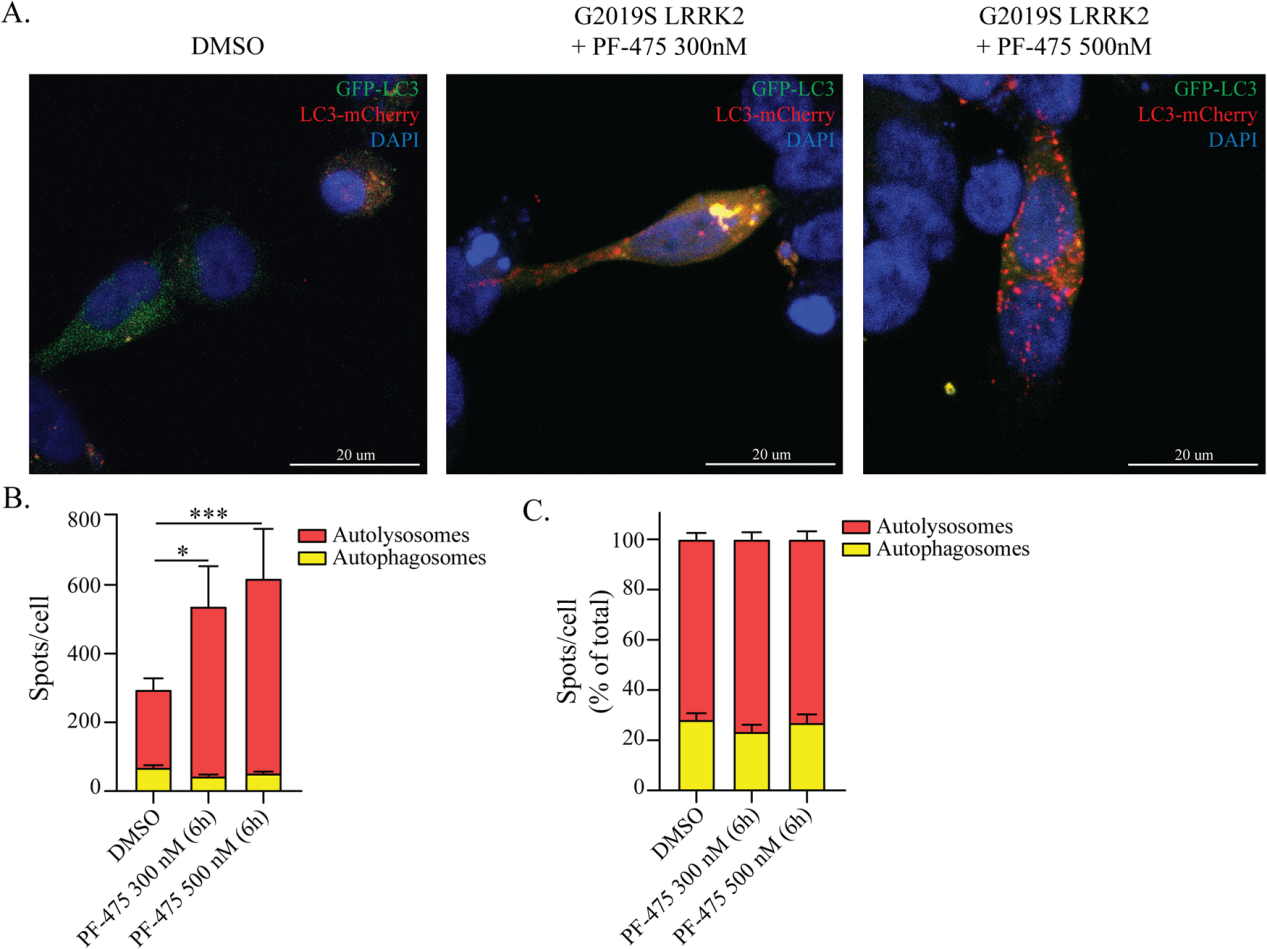
LRRK2 inhibition promotes the formation of autolysosomes. **a** G2019S-LRRK2 cells were transfected with the GFP-LC3-mCherry reporter and visualized with confocal microscopy. **b** The number of autophagosomes and autolysosomes was determined and revealed a significant increase in fusion after treatment with PF-475 at both 300 nM and 500 nM concentrations (*n* = 4). **c** The relative proportion of autophagic vesicles in each cell line was calculated and revealed no significant differences in the velocity of the autophagic flux (*n* = 4). Data are means ± SEM of four independent experiments and analysis conducted on ~100 cells per group in each experiment. *p < 0.05, ***p < 0.001, two-way ANOVA followed by Bonferroni’s post-hoc test.

Having observed positive effects of PF-475 on autophagosome number, lysosome morphology and functionality, we next asked whether the dynamic handling of autophagic vesicles could be modulated (Fig. 6a). We transfected G2019S-LRRK2 cells with the GFP-LC3-mCherry reporter construct and the number of autolysosomes was enhanced by PF-475 (Fig. 6b), without an effect on the velocity of the flux (Fig. 6c). The increase in autolysosomes is likely to be underestimated as it is probable that many autolysosomes are quickly dismantled by the process, making them undetectable in this assay.

Collectively, these data demonstrate that inhibition of G2019S-LRRK2 kinase activity rescues lysosomal abnormalities, promotes autolysosome formation and increases lysosomal activity.

### PF-475-mediated reduction of intracellular pS129-aSyn depends on autolysosome formation

Our results indicate impaired lysosome function and accumulation of intracellular pS129-aSyn in G2019S-LRRK2. The LRRK2 substrate Rab10^18,43^ localizes to autophagosomes and modulates the ALP^44,45^, directly connecting LRRK2 kinase activity to autophagy. To assess this, we nucleofected WT-Rab10 or constitutively active Q68L-Rab10. Endogenous pT73-Rab10 is enhanced by LRRK2 overexpression, with G2019S-LRRK2 exerting a stronger effect (Supplementary Fig. S5a). In addition, PF-475 concentration-dependently reduces pT73-Rab10 in G2019S-LRRK2 cells (Supplementary Fig. S5b). Both WT-Rab10 and Q68L-Rab10 are correctly phosphorylated at the LRRK2 residue T73, albeit not differently in relative magnitude (Supplementary Fig. S5c). Since LRRK2-dependent phosphorylation is suggested to prevent the interaction of Rab10 with its effectors^46^, we reasoned that a GTP-locked Rab10 might mimic the effect of PF-475 on pS129-aSyn. However, expression of neither WT-Rab10 or Q68L-Rab10 modified the number of pS129-aSyn inclusions (Supplementary Fig. S5d, e). Thus, we conclude that the reduction of inclusions depends on LRRK2 kinase activity but not on Rab10 function.

**Fig. 7:**
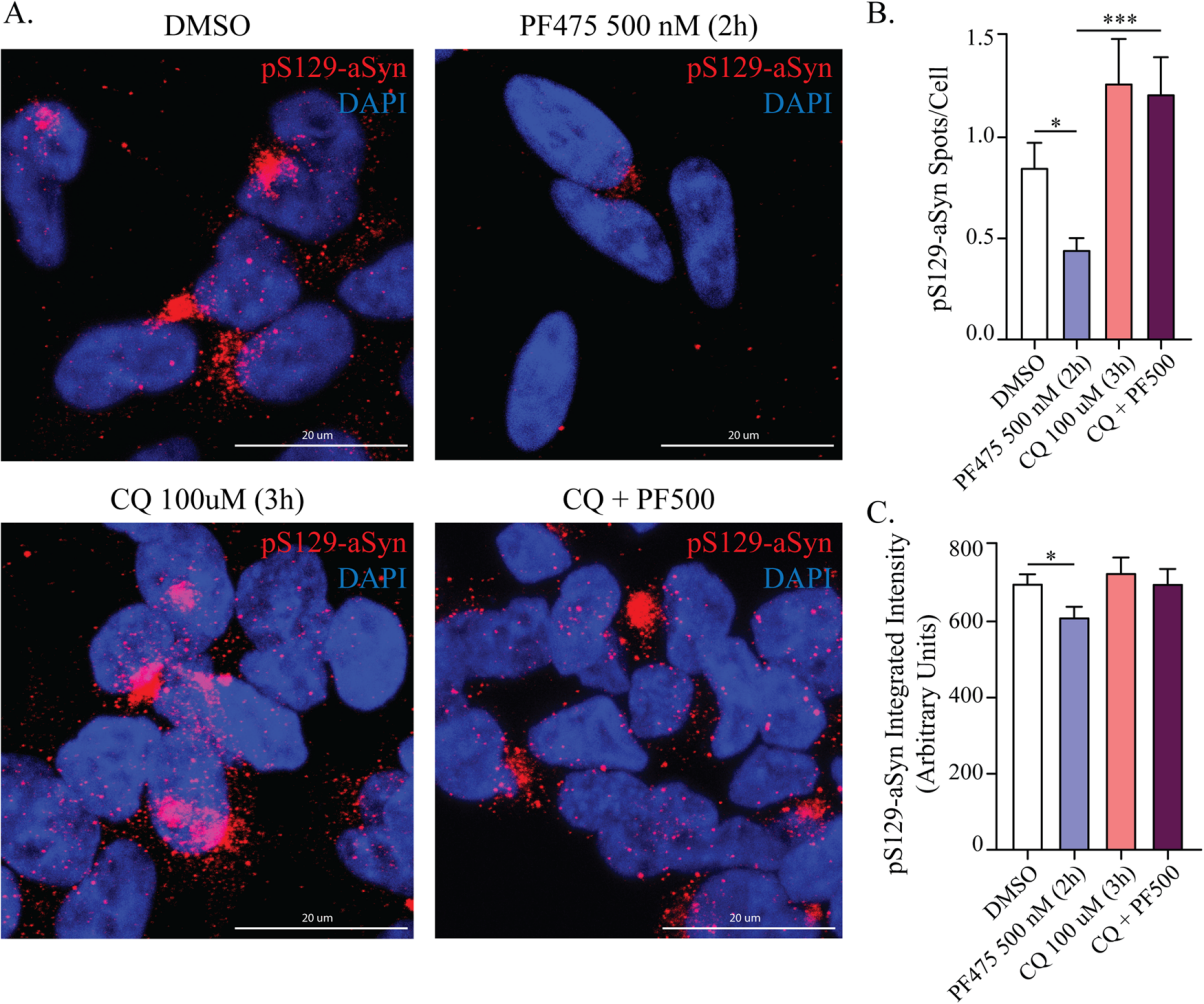
Blockade of autophagosome-lysosome fusion with CQ prevents PF-475-dependent reduction of pS129-aSyn inclusions. **a** G2019S-LRRK2 cells were either treated with PF-475 (500 nM, 2 h), CQ (100 µM, 3 h) or their combination, before processing for pS129-aSyn immunostaining and confocal microscopy. **b** The application of PF-475 reduced the number of pS129-aSyn inclusions, while CQ alone did not elicit significant effects. However, when CQ was applied before kinase inhibition, PF-475 lost its efficacy in reducing intracellular inclusion number (*n* = 3). **c** Similarly, PF-475 alone confirmed its ability in reducing pS129-aSyn signal intensity, while pre-treatment with CQ prevented this effect (*n* = 3). Data are means ± SEM of three independent experiments and analysis conducted on 700–1000 cells per group in each experiment. *p < 0.05, ***p < 0.001, one-way ANOVA followed by Bonferroni’s post-hoc test.

At this point, we have found that LRRK2 kinase inhibition promotes the clearance of autophagosomes and pS129-aSyn accumulation, while promoting lysosomal activity. A critical step for the delivery of cargos to lysosomes for degradation is the fusion between autophagosomes and lysosomes. This step is also required to clear autophagosomes. Thus, we then sought to determine if the effect of PF-475 on pS129-aSyn inclusions could directly depend on the autophagosome/lysosome fusion (which leads to autolysosome formation). Since CQ inhibits this step^47^, we applied CQ (100 µM, 3 h) to G2019S-LRRK2 cells prior to exposure to PF-475 (500 nM, 2 h), then processed the cells for immunofluorescence and confocal microscopy (Fig. 7a). We confirmed the reduction of pS129-aSyn after application of PF-475 alone. CQ alone induced a non-significant trend towards an increased number of inclusions. Importantly, PF-475 in CQ-treated cells lost its efficacy and did not alter the number of pS129-aSyn inclusions (Fig. 7b) or signal intensity (Fig. 7c).

These experiments demonstrate that PF-475 reduces pS129-aSyn inclusions by acting on the fusion step, with its upstream blockade completely preventing the positive effect of LRRK2 kinase inhibition.

## Discussion

G2019S-LRRK2 neuroblastoma cells display ALP abnormalities impacting on lysosomal activity and clearance of endogenous pS129-aSyn. Enhanced cellular levels of WT-LRRK2 also affect this pathway, however cells display strong proteolytic activity and no detectable pS129-aSyn inclusions, suggesting a potential compensatory effect. Consistently, ALP gene expression appears distinctly modulated in WT- and G2019S-LRRK2. Thus, it can be speculated that enhanced LRRK2 levels per se induce variations in this pathway, but the G2019S mutation leads to specific functional changes. Nevertheless, high LRRK2 expression levels bear an increase in the absolute kinase activity at the cellular level, possibly accounting for some of the differences we observe in WT-LRRK2 cells. Of note, our WT-LRRK2 cells display much stronger LRRK2 expression (consistent with the appearance of some phenotypes); however, (a) the relative activation of the kinase is still enhanced by G2019S, and (b) these cells do not accumulate pS129-aSyn, indicating mutation-specific dysregulation independent of expression levels.

It is established that LRRK2 plays a prominent role in autophagy, endosomal and lysosomal systems, but it is unclear which steps of the process are specifically modulated. In contrast to our results, G2019S-LRRK2 has been shown to cause increased basal autophagy and autolysosome formation in patient-derived fibroblasts. However, this was not linked to a protective mechanism but rather related to an increase in cytotoxicity^23^. Nevertheless, we report consistent findings such as increased LC3B-II levels (i.e. increase in autophagosomes) and lysosome defects^23^. The different cellular models employed might explain this discordance.

On a similar note, several studies focused on a specific step of the autophagic machinery. The overlap in LRRK2, ERK1/2 and mTOR signaling pathways served as a rationale to investigate the role of LRRK2 in autophagy initiation and autophagosome formation^13–15^. Results were contrasting and it is not currently possible to draw a definitive conclusion on whether LRRK2 kinase activity represses or enhances autophagy initiation. As mentioned above, differences in cell models utilized could underlie this discordance and it is plausible that a cell type-specific regulation exists.

Our study did not address these issues, but instead we characterized the effects of PD-linked G2019S-LRRK2 on the ALP with the specific goal of linking them directly to clearance of endogenous pS129-aSyn.

LRRK2 modulates aSyn neuropathology in a kinase-dependent manner and PD mutations worsen/sensitize to aSyn toxicity^21,29,30,40^. On the other hand, autophagy impairment can play a causative role in aSyn accumulation and nigral neurodegeneration^48–51^. In addition, alterations in autophagy-lysosome markers are found in PD brain areas affected by Lewy pathology^52,53^. However, no studies to date report an experimental demonstration that these phenomena are directly linked. Here, we report that not only does G2019S-LRRK2 cause defects in the ALP in parallel to pS129-aSyn accumulation, but also that the formation of autolysosomes is required for kinase-dependent pS129-aSyn clearance. Lastly, we dissect the modulation of aSyn inclusion burden from cytotoxicity, as cell viability is unchanged in G2019S-LRRK2 cells and not modified by kinase inhibition in this model. The role of protein aggregates in cytotoxicity and cell survival is still highly debated^54^. The data collected in our model, in which it is the endogenous aSyn that accumulates, indicate that these inclusions are not overtly harmful to the cells. Of note, these inclusions are likely an immature form of aSyn accumulation as they are readily reduced by 2 h of LRRK2 kinase inhibition. This suggests that these species are mostly soluble (i.e. not resembling a Lewy body). The slower growth rate, on the other hand, could be a consequence of a milder cellular damage produced by pS129-aSyn, but autophagy has profound consequences on cell proliferation and growth^55^. Thus, it is not possible to dissect which process mostly contributes to the growth deficit, especially because in our cell model the two phenotypes are tightly intertwined.

Our results per se hint that reduction in lysosome function underlies pS129-aSyn accumulation, rather than an overall stalling of autophagy, with kinase inhibition promoting autolysosome formation. Thus, we took advantage of the recent clarification that CQ specifically inhibits the fusion between autophagosomes and lysosomes^47^ to further clarify the cellular mechanism in LRRK2 cells. Consistently, upstream blockade of this step with CQ completely prevents the reduction in pS129-aSyn inclusions operated by PF-475, supporting the fusion step as the target of LRRK2 kinase activity and indicating it is required for clearance of pathologic aSyn. We applied CQ at a concentration that does not majorly impact lysosomal pH in the timeframe of our analyses; however, it is reported to affect the organization of the Golgi apparatus and the endosomal system^47^. This result also indicates that the increase in pS129-aSyn signal observed in G2019S-LRRK2 cells is not due to a simple increase in aSyn phosphorylation (possibly caused by the increase kinase activity of LRRK2^56^). Indeed, if that was the case, the application of CQ would have not been capable of preventing the reduction in pS129-aSyn immunosignal operated by PF-475.

We cannot exclude the lysosomal phenotypes we observe are independent from these processes; however, we find that the upstream initiation of autophagy is not affected and is not modified by LRRK2 kinase inhibition. This is consistent with active Rab10 being ineffective in pS129-aSyn clearance, as it is mostly involved in the induction of autophagy and autophagosome formation^44,45^. Nevertheless, it has been recently reported that LRRK2 is recruited onto stressed lysosomes, where it targets its substrates Rab8a and Rab10 and maintains homeostasis via a protective mechanism^16^. However, this appears to be a somewhat distinct process, as the first insult is different (direct lysosome stress vs LRRK2 mutation) and the involvement of WT vs PD-mutant LRRK2. Despite not being directly relatable, this study and our results are concordant in indicating a relevant role of LRRK2 in lysosome biology (and possibly endocytosis), with specific mechanistic differences that depend on the context (physiology vs pathogenesis).

Interestingly, other neurodegenerative diseases have been linked to autophagy-lysosome dysfunction mediating accumulation of protein aggregates^42,57,58^. Specifically, mutant Huntingtin has been reported to inhibit autophagosome-lysosome fusion in Huntington’s disease^59^.

In our study we specifically focused on macroautophagy as, in our cell models, it revealed to be the major affected mechanism, with consistent responses to kinase inhibition. Nevertheless, CMA has also been profoundly implicated in LRRK2 biology and aSyn pathology^20,21,48^. Our results do not exclude an involvement of CMA and the possibility that parallel pathways might lead to similar cellular consequences. This hypothesis finds further support from the notion that (macro)autophagy and CMA are functionally related and variations in one cause compensatory changes in the other^60,61^. In addition, we report that ~35% of inclusions are found in LC3B-positive autophagosomes. Indeed, LRRK2 has direct roles on lysosome biology and function^16^ that could “bypass” upstream steps of macroautophagy (consistent with a role in CMA). Nevertheless, promotion of ALP upon kinase inhibition is sufficient to strongly reduce the inclusion burden, while requiring correct fusion events.

LRRK2 kinase inhibitors have been developed as a disease-modifying therapy based on the etiological involvement of increased kinase activity in *Lrrk2* PD patients^38^. Preclinical models confirmed their potential in rescuing toxic effects of mutant LRRK2^62^, providing rationale for clinical trials in familial *Lrrk2* PD. However, recent evidence demonstrated that LRRK2 silencing or kinase inhibition are also effective against aSyn neuropathology and toxicity^29,30,40,63^. Importantly, endogenous LRRK2 is overactive in iPD and non-LRRK2 animal models^64^ extending their potential application. A clinical trial is ongoing to evaluate a LRRK2 kinase inhibitor in PD patients with and without LRRK2 mutations (ClinicalTrials.gov identifier: NCT04056689). Thus, it is of paramount importance to understand how the inhibitors work from the mechanistic points of view, also in consideration of their peripheral side effects^62^.

Future work will be directed towards distinguishing the role of endogenous LRRK2 and the effect of point mutations on these pathways, utilizing the evidence presented here to direct research efforts with increased efficacy.

## Materials and methods

Extended details about the methodologies employed and the materials utilized can be found in the Supplementary Materials and Methods.

### Cell cultures, drug treatments, transfection and nucleofection

SH-SY5Y neuroblastoma cell lines stably overexpressing wild-type (WT) or G2019S-LRRK2 were cultured as previously described (herein referred to as WT-LRRK2 and G2019S-LRRK2 cells)^33^ and were derived as monoclonal cells from a parental culture^65^. The LRRK2 kinase inhibitor PF-06447475 (herein, PF-475)^40^ was dissolved in DMSO and applied to cultured cells for 2 h or 6 h, with 0.1% DMSO as vehicle control.

Chloroquine (CQ; 100 µM, 3 h) was used to block to the fusion of autophagosomes with lysosomes and to evaluate the autophagic flux^47^.

The GFP-LC3-mCherry reporter construct was transfected using FuGene HD (Promega) to analyse the number of autolysosomes. Rab10-RFP constructs were nucleofected using the 4D-Nucleofector^TM^ X unit (Lonza).

### Autophagy gene expression array

SH-SY5Y, WT- and G2019S-LRRK2 cells were lysed and mRNA levels assessed using the RT2 Profiler PCR Array (PAHS-084Z) on a CFX96 Touch™ Real-Time PCR Detection System (BioRad).

### Western blotting and ProteinSimple® WES

For traditional Western blotting (WB), lysates were loaded onto a 4–12% SDS-PAGE gel and then transferred onto polyvinylidene difluoride membranes (BioRad). Chemiluminescence images were acquired using Chemidoc Touch (BioRad).

Automated capillary electrophoresis was carried out on the ProteinSimple® WES system following the manufacturer’s instructions, as previously described^66^.

### Immunofluorescence, confocal imaging and image analyses

Cells fixed in 4% paraformaldehyde (PFA) were stained with primary and relative secondary antibodies, and imaged on a Leica SP8-X confocal laser scanning microscope.

### Lysotracker Deep Red and DQ-Red-BSA staining

The Lysotracker Deep Red dye (Molecular Probes, L12492) and the fluorescent DQ-Red-BSA^36^ dye (Molecular Probes, D12051) were used to quantify lysosome morphology and proteolysis, following the manufacturer’s instructions.

### Statistical analyses

Statistical analyses were performed using GraphPad Prism 8. One-way ANOVA or two-way ANOVA were used in experiments comparing 3 or more groups, followed by Bonferroni’s post-hoc test for pairwise comparisons. With two experimental groups, the unpaired two-tailed Student’s *t*-test was utilized. Threshold for significance was set at *p* < 0.05. All experiments were performed in a minimum of three independent biological replicates.

## Supplementary information

Supplemental Material and Methods

Supplemental Table 1

Supplemental Table 2

Supplemental Figure 1

Supplemental Figure 2

Supplemental Figure 3

Supplemental Figure 4

Supplemental Figure 5
