# Supplementary material for: Kinase inhibition of G2019S-LRRK2 enhances autolysosome formation and function to reduce endogenous alpha-synuclein intracellular inclusions
Source: Cell Death Discov. 2020 Jun 8;6:45. doi: 10.1038/s41420-020-0279-y (PMC7280235; doi:10.1038/s41420-020-0279-y)
Supplement: Supplementary file 1 — Supplemental Material and Methods [file 41420_2020_279_MOESM1_ESM.docx]

**Kinase inhibition of G2019S-LRRK2 restores autolysosome formation and function to reduce endogenous alpha-synuclein intracellular inclusions**

Julia Obergasteiger^a,*^, Giulia Frapporti^a,*,†^, Giulia Lamonaca^a^, Sara Pizzi^a^, Anne Picard^a^, Alexandros A. Lavdas^a^, Francesca Pischedda^b^, Giovanni Piccoli^b^, Sabine Hilfiker^c^, Evy Lobbestael^d^, Veerle Baekelandt^d^, Andrew A. Hicks^a^, Corrado Corti^a^, Peter P. Pramstaller^a,e,f^ and Mattia Volta^a,#^

**Supplementary materials and methods**

*Cell culture and drug treatment*

SH-SY5Y neuroblastoma cell lines stably overexpressing wild-type (WT) or G2019S-LRRK2 were previously described (herein referred to as WT-LRRK2 and G2019S-LRRK2 cells)^1, 2^. Control SH-SY5Y cells were maintained in DMEM GlutaMAX medium, supplemented with 10% fetal bovine serum (FBS) and 1% penicillin/streptomycin. Recombinant LRRK2 cells were cultured in DMEM GlutaMAX, 15% FBS, 1% non-essential amino acids, 50µg/ml gentamycin (Gibco) and 200µg/ml hygromycin B (Invitrogen). All cells were incubated at 37°C with 5% CO_2_.

The LRRK2 kinase inhibitor PF-06447475 (herein, PF-475)^3^ was dissolved in DMSO and applied to cultured cells for 2h or 6h, with 0.1% DMSO used as vehicle control.

Chloroquine (CQ; 100µM, 3h) was used to block to the fusion of autophagosomes with lysosomes and to evaluate the autophagic flux^4^.

*Transfection and Nucleofection*

Transfection of the GFP-LC3-mCherry reporter construct was carried out using FuGene HD (Promega) with 800ng of DNA (ratio 5.2:1) when 50%-60% confluent, then incubated at 37°C/5% CO_2_ for 48h and then fixed with 4% PFA.

GFP fluorescence is pH-sensitive, thus, in the cytosol and in autophagosomes, both GFP and mCherry fluorophores are active and both green and red fluorescence are detected. Upon fusion of the autophagosome with the lysosome and formation of the autolysosome, the pH turns acidic and quenches the GFP signal.

Images were acquired with the confocal microscope and analysed with CellProfiler, determining the turnover from autophagosomes to autolysosomes (red/green ratio).

Expression of Rab10-RFP constructs (kindly provided by Prof. Sabine Hilfiker, Rutgers University) was achieved via electroporation using the 4D-Nucleofector^TM^ X system (Lonza). G2019S-LRRK2 cells were nucleofected using the SF Cell Line 4D-Nucleofector^TM^ X kit. Nucleofection was carried out according to the manufacturers protocol. Briefly, 200 000 cells were resuspended in the nucleofection solution containing 800ng plasmid DNA and incubated for 10min after nucleofection. Then cells were plated in complete media on coverslips and analyzed after 48h.

*Live/Dead assay*

The ReadiDrop 7-aminoactinomycin D (7-AAD) cell viability dye (BioRad) was used to assess the number of live and dead cells. SH-SY5Y, WT- and G2019S-LRRK2 cells, or G2019S LRRK2 treated with PF-475 (300 and 500nM, 6h) were detached with Trypsin, collected in a tube, washed with PBS and then incubated for 5 min in the dark in 500ml PBS containing one drop of the cell viability dye. Cell suspension was passed through a 70µm cell strainer, transferred to a tube for fluorescence-activated cell sorting (FACS) and vortexed. Cells were acquired on the S3e™ Cell Sorter (BioRad). Data were analyzed using FlowJo software (BD). Cells were analysed in FSC-A and SSC-A to morphologically identify populations of interest and exclude debris. Single cells were distinguished from cell aggregates by plotting FSC-A against FSC-W and a gate was set, to exclude aggregates from analysis. Single cells were analyzed in FSC-A and FL4-A as a gate strategy to identify the live-cell population. Data are presented as percentage of 7-AAD negative cells.

*ATPlite cell growth assay*

SH-SY5Y, WT- and G2019S-LRRK2 cells were plated in triplicates on 4 replica 96-well plates (5000 cells/well). On day 1 to day 4 one replica plate was used for analysis of ATP levels using the ATPlite 1step Luminescence Assay kit (Perkin Elmer). The assay was carried out using the manufacturer’s instructions. Briefly, medium was removed and cells were washed with PBS. Then, cells were lysed in 60µl of reconstituted ATPlite reagent and shaken for 2min in the dark. Next, 30µl of the cell lysate were transferred to a white 96-well half-area plate and luminescence was measured on the Envision system (Perkin Elmer). Results are expressed as fold-change with respect to day 1.

*Autophagy gene expression array*

SH-SY5Y, WT- and G2019S-LRRK2 cells were lysed in RLT Plus buffer containing 1% β-mercaptoethanol. Total RNA was extracted using the RNeasy Plus Mini Kit (Qiagen). The RNA concentration was quantitated with QuantiFluor® RNA System (Promega) and quality was checked on a capillary electrophoresis instrument (Experion, BioRad). First strand cDNA was synthesized from 500ng total RNA using the RT2 First Strand Kit (Qiagen) and processes according to the manufacturer´s protocol. Briefly, cDNA was mixed with RT2 SYBR® Green qPCR Mastermix (Qiagen) and dispensed to each well of the RT2 Profiler PCR Array (PAHS-084Z). Quantitative PCR was performed in a CFX96 Touch™ Real-Time PCR Detection System (BioRad). Analysis was carried out with the webtool provided by Qiagen (<https://www.qiagen.com/it/shop/genes-and-pathways/data-analysis-center-overview-page/>). An Excel file with CT values was uploaded to the website, samples assigned to control and test groups and CT values normalized based on the geometric mean of five housekeeping genes (*ACTB*, *B2M*, *GAPDH*, *HPRT1* and *RPLP0*). The webtool calculates fold change using the Delta Delta C_T_ method.

*Western blotting and ProteinSimple® WES*

Cells were lysed in RIPA (Sigma-Aldrich) containing protease and phosphatase inhibitors (cOmplete and PhosSTOP; Roche). Lysates were sonicated for 10sec and centrifuged at 10 000xg for 10min at 4°C. For traditional Western blotting (WB), lysates were heated in sample buffer (LDS sample buffer, NuPAGE) containing 50mM dithiothreitol (95°C, 5min), loaded onto a 4-12% SDS-PAGE gel and then transferred onto polyvinylidene difluoride membranes (BioRad). Primary antibodies were: anti-LRRK2 1:20 000 (Abcam, ab172378), anti-pS935-LRRK2 1:1000 (Abcam, ab172382), anti-pS1292-LRRK2 1:1000 (Abcam, ab206035), anti-LC3 1:1000 (Cell Signaling Technologies, 3868), anti-β-actin 1:6000 (Sigma Aldrich, A-5316). Chemiluminescence images were acquired using Chemidoc Touch (BioRad) and relative band intensity calculated using ImageLab software (BioRad).

Automated capillary electrophoresis was carried out on the ProteinSimple® WES system. Lysates were prepared as above and then run on the system following the manufacturer’s instructions. Lysate concentration: 1.5mg/ml; antibody concentrations: anti-β-actin (Sigma, A2228) 1:50, anti-Rab10 (Abcam, ab181367) 1:25, anti-pT73-Rab10 (Abcam, ab230261) 1:25. In the electropherograms, the area under each peak corresponding to the protein of interest was calculated.

*Immunofluorescence, confocal imaging and image analyses*

Cells were fixed in 4% paraformaldehyde (PFA), then permeabilized, blocked with bovine serum albumin and incubated with primary antibodies overnight at 4°C. The following day, after washing, cells were incubated with secondary fluorescent antibodies (2h, room temperature), washed and mounted with DAPI. Primary antibodies: rabbit anti-LC3B 1:2000 (Molecular Probes, L10382), mouse anti-pS129-aSyn 1:2000 (Abcam, ab184674). Secondary antibodies: [Donkey anti-Rabbit Alexa Fluor 488](https://www.thermofisher.com/antibody/product/A21206) (A-21206); Donkey anti-Mouse Alexa Fluor 555 (A31570). Visualization was performed using a Leica SP8-X confocal laser scanning microscope equipped with an oil immersion 63X objective.

Projected stacks were analysed with custom pipelines in CellProfiler^5^ to quantify the number of fluorescent puncta and their integrated intensity. Pipelines are available upon request.

*Lysotracker Deep Red and DQ-Red-BSA staining*

To investigate lysosome morphology, we utilized the Lysotracker Deep Red dye (Molecular Probes, L12492) following the manufacturer´s instructions. Briefly, cells were incubated with Lysotracker for 20min, then DAPI for live imaging (Invitrogen, R37605) was added and cells were visualized live on the confocal microscope in an environmental chamber, using resonant scanning at 8KHz to minimize phototoxicity. Stacks were reconstructed in Imaris software (BitPlane) to 3D structures, and the number and diameter of the lysosomes was quantified by the software.

To study the lysosome proteolytic activity, the fluorescent DQ-Red-BSA^6^ dye (Molecular Probes, D12051) was used following manufacturer´s instructions. Briefly, cells were incubated with the dye for 120min, then DAPI for live imaging was added and visualization performed with confocal microscopy. Projected stack images were then analysed with CellProfiler to quantify the number of DQ-Red-BSA spots.

1. Obergasteiger J, Uberbacher C, Pramstaller PP, Hicks AA, Corti C, Volta M. CADPS2 gene expression is oppositely regulated by LRRK2 and alpha-synuclein. *Biochemical and biophysical research communications* 2017, **490**(3)**:** 876-881.

2. Vancraenenbroeck R, De Raeymaecker J, Lobbestael E, Gao F, De Maeyer M, Voet A*, et al.* In silico, in vitro and cellular analysis with a kinome-wide inhibitor panel correlates cellular LRRK2 dephosphorylation to inhibitor activity on LRRK2. *Frontiers in molecular neuroscience* 2014, **7:** 51.

3. Daher JP, Abdelmotilib HA, Hu X, Volpicelli-Daley LA, Moehle MS, Fraser KB*, et al.* Leucine-rich Repeat Kinase 2 (LRRK2) Pharmacological Inhibition Abates alpha-Synuclein Gene-induced Neurodegeneration. *The Journal of biological chemistry* 2015, **290**(32)**:** 19433-19444.

4. Mauthe M, Orhon I, Rocchi C, Zhou X, Luhr M, Hijlkema KJ*, et al.* Chloroquine inhibits autophagic flux by decreasing autophagosome-lysosome fusion. *Autophagy* 2018, **14**(8)**:** 1435-1455.

5. Kamentsky L, Jones TR, Fraser A, Bray MA, Logan DJ, Madden KL*, et al.* Improved structure, function and compatibility for CellProfiler: modular high-throughput image analysis software. *Bioinformatics* 2011, **27**(8)**:** 1179-1180.

6. Frost LS, Dhingra A, Reyes-Reveles J, Boesze-Battaglia K. The Use of DQ-BSA to Monitor the Turnover of Autophagy-Associated Cargo. *Methods in enzymology* 2017, **587:** 43-54.
