# Supplementary material for: Kinase inhibition of G2019S-LRRK2 enhances autolysosome formation and function to reduce endogenous alpha-synuclein intracellular inclusions
Source: Cell Death Discov. 2020 Jun 8;6:45. doi: 10.1038/s41420-020-0279-y (PMC7280235; doi:10.1038/s41420-020-0279-y)
Supplement: Supplementary file 2 — Supplemental Table 1 [file 41420_2020_279_MOESM2_ESM.docx]

| **Symbol** | **Fold Change (comparing to SH-SY5Y)** | |
| --- | --- | --- |
|  | **WT LRRK2** | **G2019S LRRK2** |
|  | **Fold Change** | **Fold Change** |
| **AKT1** | 1.4747 | 1.1772 |
| **AMBRA1** | 1.076 | 1.1866 |
| **APP** | 1.1437 | 1.1345 |
| **ATG10** | 0.9823 | 0.7218 |
| **ATG12** | 1.015 | 0.9341 |
| **ATG16L1** | 1.2251 | 0.6987 |
| **ATG16L2** | 0.9191 | 0.5776 |
| **ATG3** | 0.8234 | 0.9559 |
| **ATG4A** | 1.0241 | 1.0386 |
| **ATG4B** | 0.9183 | 1.1075 |
| **ATG4C** | 0.7711 | 0.517 |
| **ATG4D** | 0.871 | 0.8158 |
| **ATG5** | 1.0617 | 1.1778 |
| **ATG7** | 1.3872 | 1.7698 |
| **ATG9A** | 1.2125 | 0.9067 |
| **ATG9B** | 0.7768 | 1.0211 |
| **BAD** | 0.9962 | 0.7481 |
| **BAK1** | 0.6167 | 0.6609 |
| **BAX** | 1.1471 | 0.9912 |
| **BCL2** | 1.721 | 1.7125 |
| **BCL2L1** | 1.1551 | 1.3189 |
| **BECN1** | 0.9207 | 1.1203 |
| **BID** | 0.762 | 0.7585 |
| **BNIP3** | 0.8081 | 0.8059 |
| **CASP3** | 1.8135 | 1.6912 |
| **CDKN1B** | 1.2985 | 0.8137 |
| **CDKN2A** | 3.7052 | 9.1833 |
| **CLN3** | 0.8196 | 0.8844 |
| **CTSB** | 2.7017 | 2.0824 |
| **CTSD** | 1.2013 | 1.0232 |
| **CTSS** | 0.4744 | 0.2127 |
| **CXCR4** | 7.6986 | 20.059 |
| **DAPK1** | 0.8166 | 0.9504 |
| **DRAM1** | 1.3401 | 0.6744 |
| **DRAM2** | 1.1283 | 1.061 |
| **EIF2AK3** | 1.4516 | 0.8169 |
| **EIF4G1** | 0.886 | 1.0238 |
| **ESR1** | 0.8725 | 0.4806 |
| **FADD** | 0.9149 | 1.1835 |
| **FAS** | 0.9578 | 1.8232 |
| **GAA** | 0.8453 | 0.8091 |
| **GABARAP** | 1.0485 | 0.8966 |
| **GABARAPL1** | 1.3248 | 0.8569 |
| **GABARAPL2** | 1.187 | 1.1665 |
| **HDAC1** | 0.8974 | 0.9478 |
| **HDAC6** | 1.336 | 0.8641 |
| **HGS** | 0.8673 | 1.1755 |
| **HSP90AA1** | 1.1748 | 1.7913 |
| **HSPA8** | 0.9984 | 1.7976 |
| **HTT** | 0.912 | 0.7051 |
| **INS** | 0.2576 | 0.3606 |
| **LAMP1** | 1.1317 | 1.0807 |
| **MAP1LC3A** | 1.2958 | 1.3162 |
| **MAP1LC3B** | 1.3364 | 1.5 |
| **MAPK14** | 1.3022 | 1.2991 |
| **MAPK8** | 1.5531 | 1.5069 |
| **MTOR** | 1.0465 | 1.0032 |
| **NFKB1** | 1.0485 | 1.0382 |
| **NPC1** | 0.938 | 0.8411 |
| **PIK3C3** | 0.7689 | 1.059 |
| **PIK3R4** | 0.8715 | 0.9327 |
| **PRKAA1** | 1.2937 | 1.164 |
| **PTEN** | 1.0761 | 1.3675 |
| **RAB24** | 0.939 | 0.8338 |
| **RB1** | 1.1194 | 0.9508 |
| **RGS19** | 0.8857 | 0.8221 |
| **RPS6KB1** | 1.0645 | 0.963 |
| **SNCA** | 0.7683 | 0.7474 |
| **SQSTM1** | 0.9791 | 1.0981 |
| **TGFB1** | 0.6876 | 0.6546 |
| **TGM2** | 2.0779 | 0.5568 |
| **TMEM74** | 0.6818 | 0.9542 |
| **TNFSF10** | 0.8986 | 0.5604 |
| **TP53** | 0.7308 | 0.9354 |
| **ULK1** | 1.0795 | 0.9113 |
| **ULK2** | 1.093 | 0.7751 |
| **UVRAG** | 1.1362 | 0.972 |
| **WIPI1** | 0.5497 | 0.3027 |

*Table 1. Relative expression changes of genes related to the autophagy-lysosome pathway in WT- and G2019S-LRRK2 cells, compared to SH-SY5Y controls.*
