# Supplementary figures and images for: Kinase inhibition of G2019S-LRRK2 enhances autolysosome formation and function to reduce endogenous alpha-synuclein intracellular inclusions
Source: Cell Death Discov. 2020 Jun 8;6:45. doi: 10.1038/s41420-020-0279-y (PMC7280235; doi:10.1038/s41420-020-0279-y)

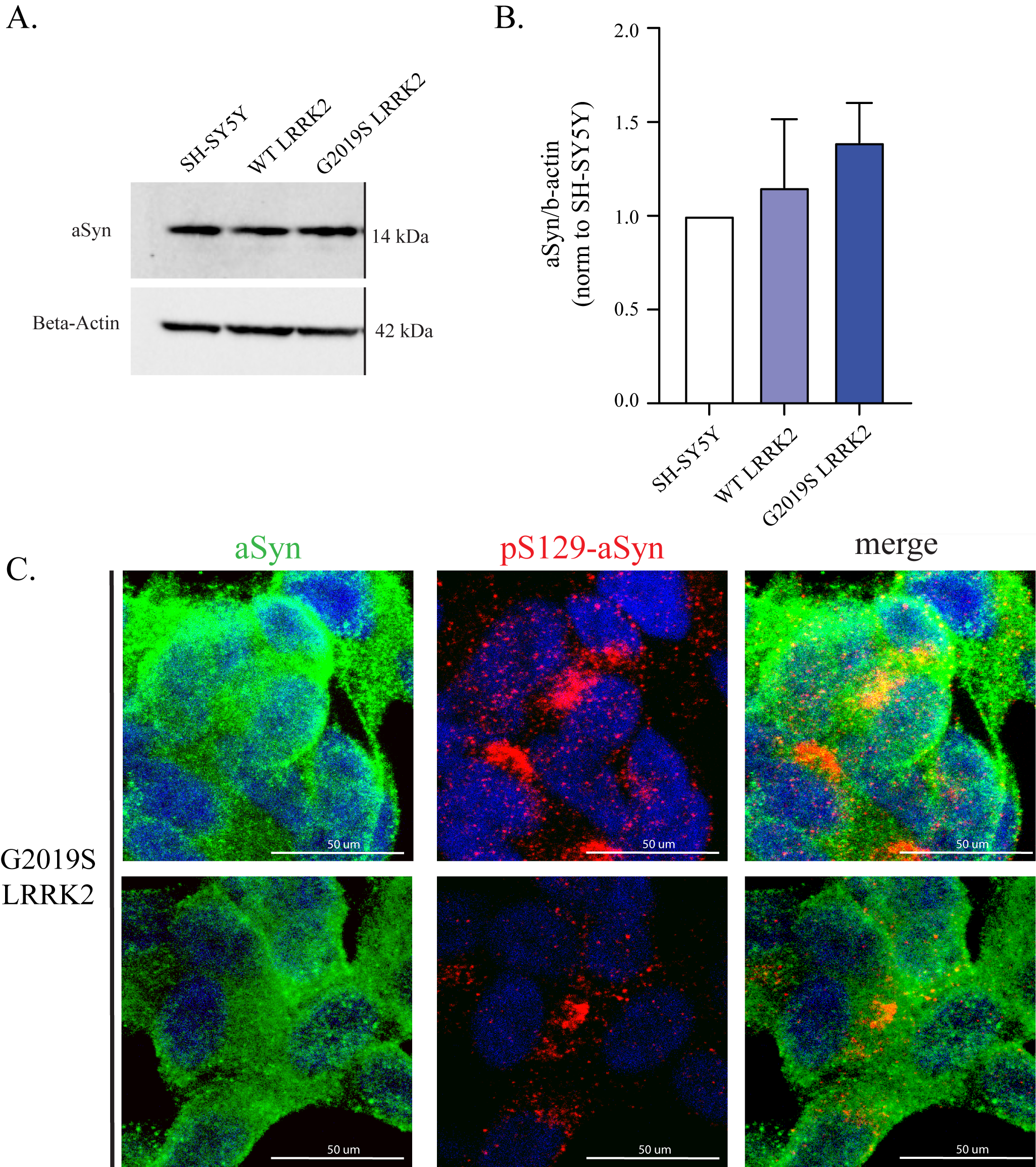

Supplement: Supplementary file 4 — Supplemental Figure 1 [file 41420_2020_279_MOESM4_ESM.tif]

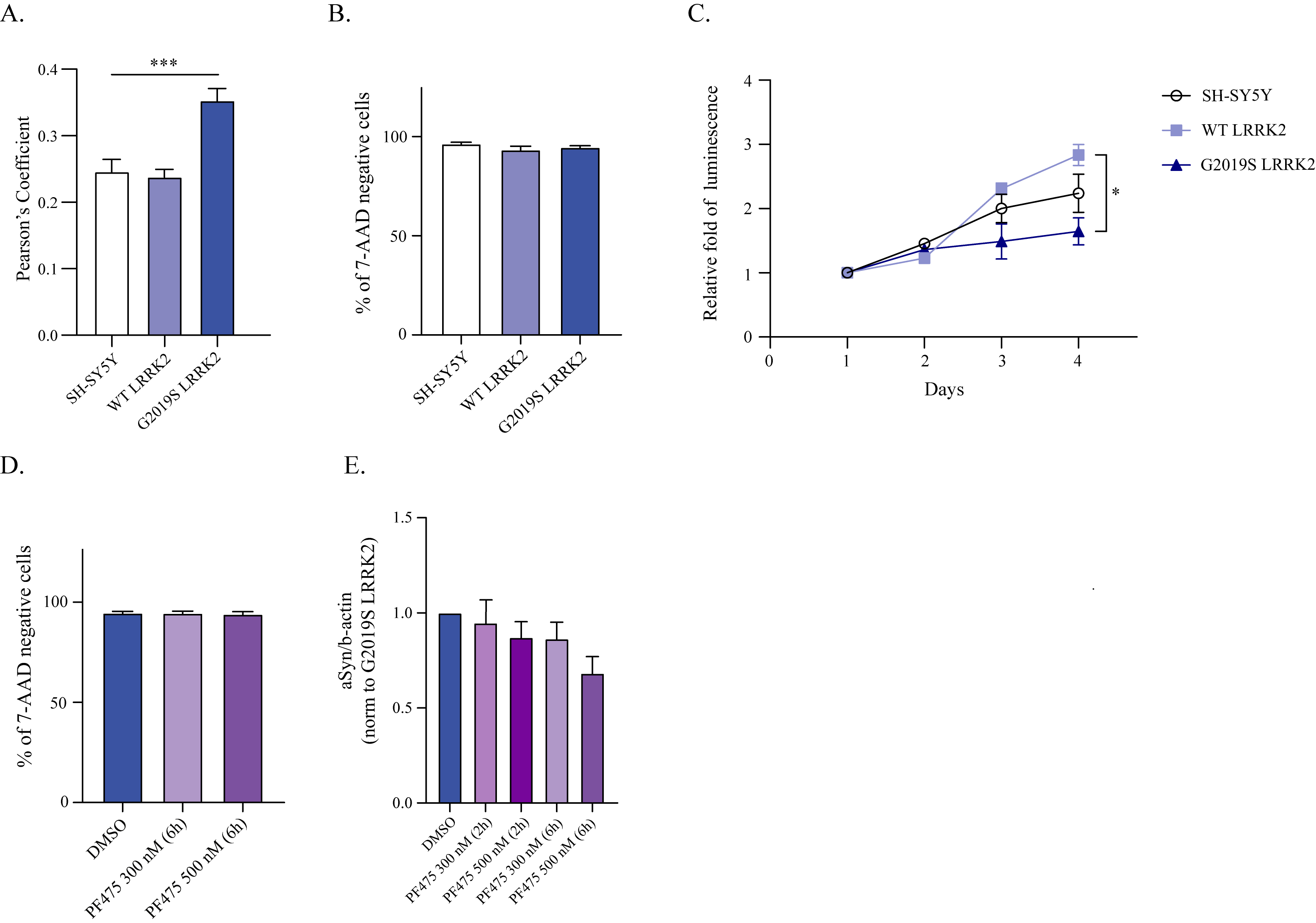

Supplement: Supplementary file 5 — Supplemental Figure 2 [file 41420_2020_279_MOESM5_ESM.tif]

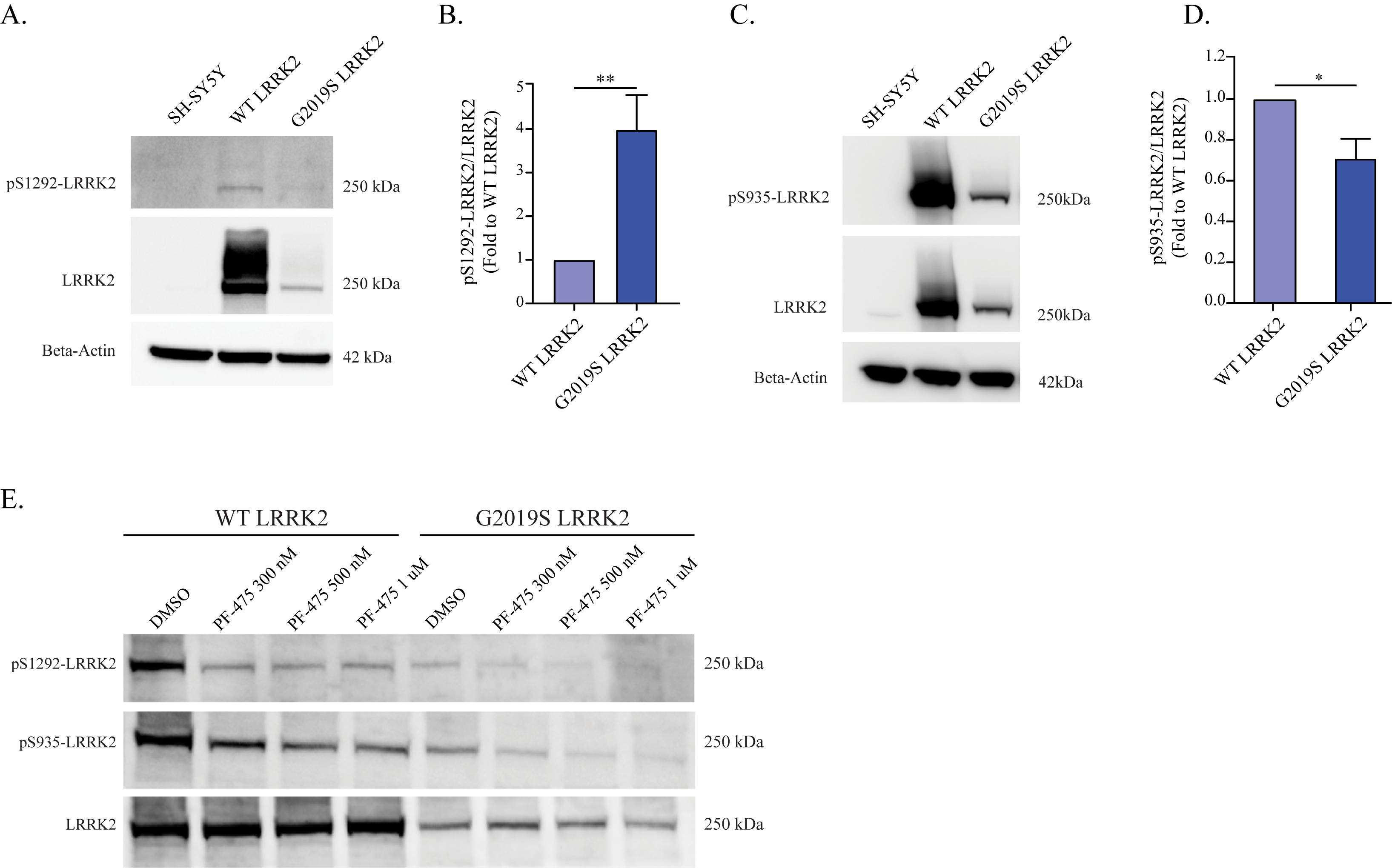

Supplement: Supplementary file 6 — Supplemental Figure 3 [file 41420_2020_279_MOESM6_ESM.tif]

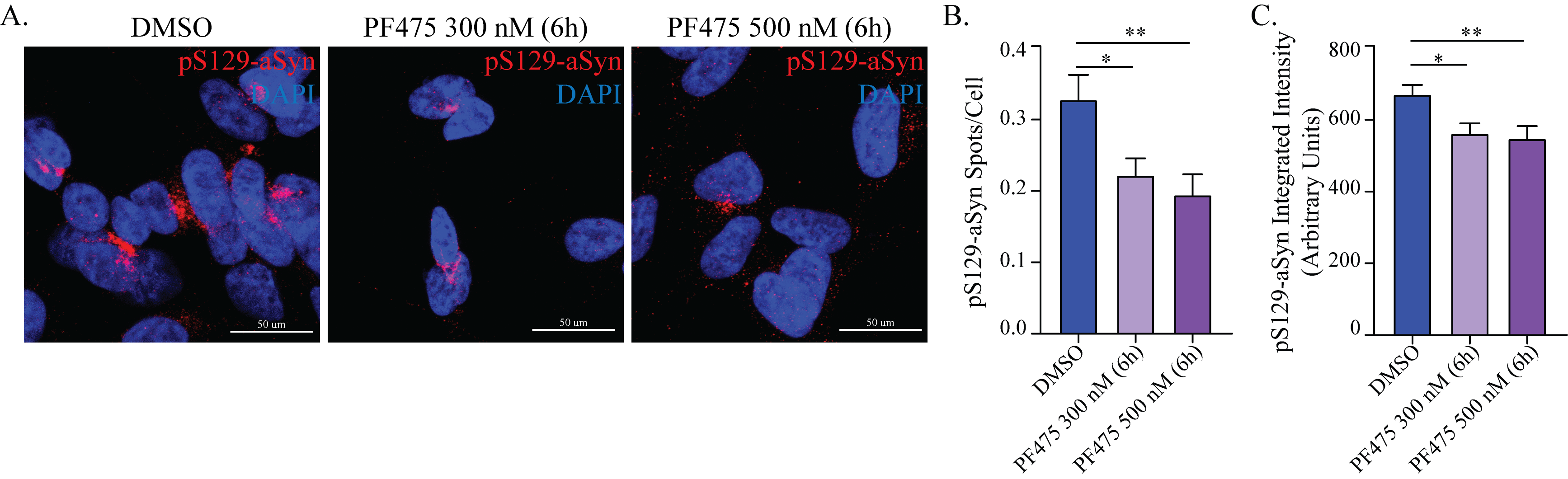

Supplement: Supplementary file 7 — Supplemental Figure 4 [file 41420_2020_279_MOESM7_ESM.tif]

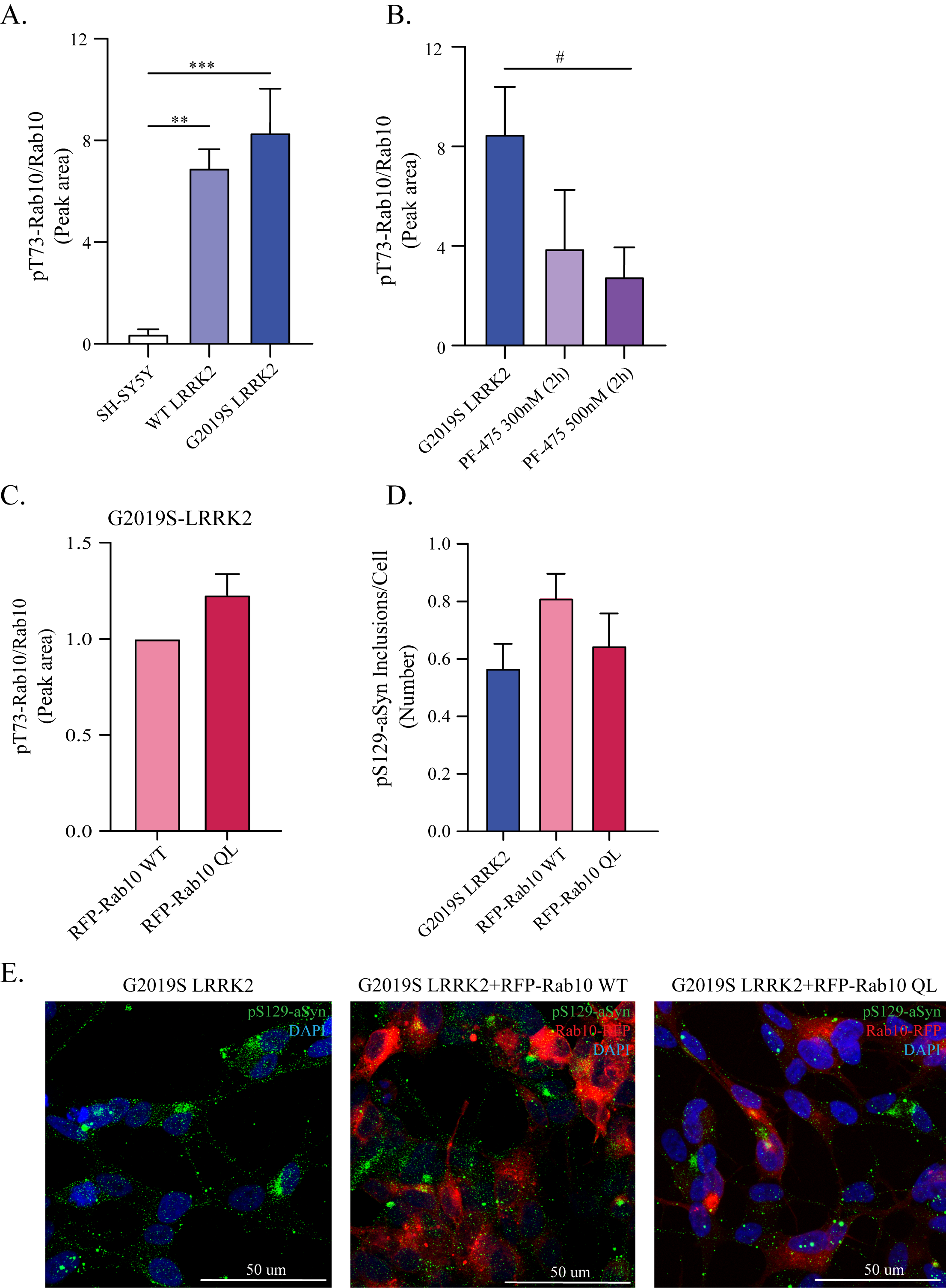

Supplement: Supplementary file 8 — Supplemental Figure 5 [file 41420_2020_279_MOESM8_ESM.tif]
